# Correlation between the cardiometabolic index and arteriosclerosis in patients with type 2 diabetes mellitus

**DOI:** 10.1186/s12872-024-03853-8

**Published:** 2024-03-27

**Authors:** Chaoyan Tang, Tianjiao Pang, Chaozhi Dang, Hui Liang, Junfeng Wu, Xiaofang Shen, Lielin Wang, Ruiqiong Luo, Haiyun Lan, Ping Zhang

**Affiliations:** Department of Endocrinology, Yulin First People’s Hospital, Sixth Affiliated Hospital of Guangxi Medical University, No.495, Education Middle Road, Yulin, 537000 Guangxi Zhuang Autonomous Region China

**Keywords:** Type 2 Diabetes Mellitus, Cardiometabolic Index, Arteriosclerosis, Cardiovascular Risk

## Abstract

**Background:**

The cardiometabolic index (CMI) is a new metric derived from the triglyceride-glucose index and body mass index and is considered a potential marker for cardiovascular risk assessment. This study aimed to examine the correlation between the CMI and the presence and severity of arteriosclerosis in patients with type 2 diabetes mellitus (T2DM).

**Methods:**

This study involved 2243 patients with T2DM. The CMI was derived by dividing the triglyceride level (mmol/L) by the high-density lipoprotein level (mmol/L) and then multiplying the quotient by the waist-to-height ratio. Multivariate logistic regression was used to analyze the correlations between the CMI and BMI blood biomarkers, blood pressure, and brachial-ankle pulse wave velocity (baPWV).

**Results:**

Patients were categorized into three groups based on their CMI: Group C1 (CMI < 0.775; *n* = 750), Group C2 (CMI: 0.775–1.355; *n* = 743), and Group C3 (CMI > 1.355; *n* = 750). Increased BMI, fasting glucose, insulin (at 120 min), total cholesterol (TC), and baPWV values were observed in Groups C2 and C3, with statistically significant trends (all trends *P* < 0.05). The CMI was positively correlated with systolic blood pressure (*r* = 0.74, *P* < 0.001). Multivariate analysis revealed that an increased CMI contributed to a greater risk for arteriosclerosis (OR = 1.87, 95%CI: 1.66–2.10, *P* < 0.001). Compared to the C1 group, the C2 group and C3 group had a greater risk of developing arteriosclerosis, with ORs of 4.55 (95%CI: 3.57–5.81, *P*<0.001) and 5.56 (95%CI: 4.32–7.17, *P*<0.001), respectively. The association was notably stronger in patients with a BMI below 21.62 kg/m² than in those with a BMI of 21.62 kg/m² or higher (OR = 4.53 vs. OR = 1.59).

**Conclusions:**

These findings suggest that the CMI is a relevant and independent marker of arteriosclerosis in patients with T2DM and may be useful in the risk stratification and management of these patients.

## Introduction

Diabetes mellitus (DM) is one of the major diseases endangering human health worldwide and is a multifactorial chronic health disease; approximately 90-95% of the population with DM has type 2 diabetes mellitus (T2DM) [1, 2]. T2DM can develop at a younger age (40 years), resulting in more years of life lost, and is also becoming more common [3]. As the prevalence of obesity increases worldwide, so does the prevalence of T2DM, which now affects more than 370 million people [4]. Diabetes is a major risk factor for cardiovascular diseases, including diabetic cardiomyopathy, atherosclerosis, myocardial infarction, and heart failure [5, 6]. There is evidence that cardiovascular and cerebrovascular accidents are the main outcomes of diabetic patients, and the pathological changes of these accidents are characterized by arteriosclerosis [7]. The risk of developing atherosclerosis is no longer limited to Western countries and is prevalent in the majority of global mortality cases. Atherosclerosis now affects a broader demographic population, including younger individuals, women, and people from diverse ethnic backgrounds [8].

The cardiometabolic index (CMI), a new metric derived from the triglyceride-glucose index and body mass index, has emerged as a potentially valuable marker for cardiovascular risk assessment [9]. Several studies have highlighted the utility of the CMI as a predictor of cardiovascular diseases in the general population [10, 11]. However, the correlation of the CMI with specific pathological features of cardiovascular disease, such as arteriosclerosis, in T2DM patients remains inadequately explored. In recent years, the CMI has been considered to have some significance in the screening of diabetes, atherosclerosis, and renal dysfunction [12, 13]. The CMI, which is determined by the waist-height ratio and the triglyceride-to-high-density lipoprotein cholesterol ratio, serves as a novel criterion for diabetes [14].

This study sought to bridge this knowledge gap by investigating the correlation between the CMI and arteriosclerosis in patients with T2DM. Establishing this relationship could enhance our understanding of cardiovascular risk stratification and potentially lead to more targeted interventions to mitigate the risk of arteriosclerosis and its consequent cardiovascular events in patients with T2DM.

## Methods and materials

### Patients

This study included patients with T2DM who were treated for the first time at the National Metabolic Management of Metabolic Center (MMC). These individuals were either newly diagnosed with diabetes or had a prior diagnosis and had been irregularly taking hypoglycemic drugs. None of the patients had used lipid-lowering drugs in the last six months. The present study included a total of 2940 patients with T2DM who received treatment for the first time from April 1, 2018, to April 30, 2023. Individuals who met the following inclusion criteria were included: (1) had T2DM and met the WHO 1999 diagnostic criteria for diabetes; and (2) were aged 18 to 75 years. The exclusion criteria were as follows: (1) the presence of acute complications of diabetes; (2) the use of oral lipid-lowering drugs and corticosteroids in the past six months; (3) the presence of acute infections, malignant tumors, or hyperthyroidism; (4) a history of coronary heart disease or stroke; and (5) incomplete data. A total of 2243 participants were ultimately included in the analysis. Informed consent was obtained from all participants, and the study was approved by The Ethics Committee of the First People’s Hospital of Yulin.

## Anthropometric, clinical, and sociodemographic parameters

Trained staff recorded each participant’s anthropometric data. Body weight and height were recorded with participants in light attire and without shoes. BMI was calculated using the formula weight (kg) divided by height squared (m²). Waist circumference was determined at the midpoint between the lowest rib and the top of the hip bone. Additionally, we calculated the waist-to-height ratio (WHtR) as waist circumference divided by height, both in centimeters. The CMI was derived by dividing the triglyceride (TG) level (mmol/L) by the high-density lipoprotein (HDL-C) level (mmol/L) and then multiplying the quotient by the WHtR according to the formula: CMI = TG/HDL-C × WHtR. Blood pressure readings were taken using an automated electronic device (OMRON HBP-1100 U) after the participant had been seated and at rest for a minimum of five minutes, with the arm in which blood pressure was measured supported at heart level. Participants also underwent brachial-ankle pulse wave velocity (baPWV) assessments using an OMRON BP-203RPE III automated device. After at least five minutes of rest, the cuffs were secured to the participants’ arms and ankles, and simultaneous measurements were taken at the brachial and tibial arteries. We defined transit time as the duration between the initial rise in brachial and tibial waveforms, and we measured the transit distance between the arm and ankle. The baPWV was calculated by dividing the transit distance by the transit time. The enrolled participants were divided into a normal baPWV group (nonarteriosclerosis group (≤ 1400 cm/s)) and an elevated baPWV group (> 1400 cm/s).

### Laboratory assays

We collected fasting venous blood samples from all participants after 10 to 12 h of fasting. Postprandial samples were obtained 120 min after a standardized meal consisting of steamed bread to evaluate glucose levels. Biochemical parameters were analyzed using a Roche Cobas 702 automatic biochemical analyzer, and the levels of serum insulin and C-peptide were assessed with an automatic glycosylated hemoglobin analyzer (SySMEX HLC-723G8).

### Statistical analysis

Normally distributed measurement data are presented as the mean ± standard deviation, while nonnormally distributed data are reported as medians and interquartile ranges. Counts are expressed as frequencies and percentages (n, %). The patient characteristics were categorized by tertiles of the CMI. To determine the trends in continuous and categorical variables, linear regression analysis and chi-square trend tests, respectively, were performed. The Spearman correlation coefficient was used to explore the relationships between the CMI and other indicators. The subgroup analyses were based on the following predetermined cutoff values: diastolic blood pressure (78.50 mmHg), systolic blood pressure (128.50 mmHg), BMI (21.62 kg/m²), hip circumference (90.75 cm), fasting glucose level (5.26 mmol/L), insulin level at 120 min (29.65 µIU/mL), and total cholesterol level (4.88 mmol/L). Multivariate logistic regression, yielding odds ratios (ORs) and 95% confidence intervals (CIs), was used to assess the independent impact of the CMI on arteriosclerosis. SPSS 27.0 was utilized for the statistical analysis, and *p* values less than 0.05 were considered to indicate statistical significance.

## Results

### Demographic and clinical characteristics by the CMI

The demographic and clinical characteristics of the included participants are shown in Table 1. In this study, we included 2,243 patients diagnosed with T2DM. Of these patients, 1,300 (58.0%) were male, and 943 (42.0%) were female. The median age of the included patients was 55 years. Patients were categorized into three groups based on their CMI: Group C1, which included patients with a CMI less than 0.775 (*n* = 750); Group C2, which included patients with a CMI ranging from 0.775 to 1.355 (*n* = 743); and Group C3, which included patients with a CMI greater than 1.355 (*n* = 750). Patients in Group C3 exhibited greater hip circumferences than did those in Groups C1 and C2 (*P* < 0.001). Additionally, Groups C2 and C3 had higher BMI values than did Group C1 (*P* < 0.001). Increased fasting glucose, insulin (at 120 min), TC and baPWV values were observed in Groups C2 and C3, with statistical significance (all trends *P* < 0.05). There was no significant difference in age or disease course across the groups (*P* = 0.237 and *P* = 0.735).

**Table 1:** Demographic and Clinical Characteristics of the Study Population According to the CMI

Variables	C1 (*n* = 750)	C2 (*n* = 743)	C3 (*n* = 750)	* P *
Gender (male/female, n)	411/339	422/321	467/283	0.003
Age (year)	54 (47, 61)	56 (49, 63)	55 (48, 62)	0.237
Duration (day)	24.00 (1.00, 85.25)	20.00 (0, 97.00)	23.50 (0, 85.00)	0.735
Diastolic blood pressure (mmHg)	77 (70, 84)	78 (71, 86)	81 (74, 88)	<0.001
Systolic blood pressure (mmHg)	127 (115, 139)	133 (119, 147)	134 (121, 146)	<0.001
BMI (kg/m^2^)	22.65 (20.70, 24.91)	24.26 (22.03, 26.53)	24.91 (22.79, 27.34)	<0.001
Hip circumference (cm)	91 (87.00, 96.00)	94 (89.00, 99.00)	90 (95.00, 99.25)	<0.001
Glucose 0 min (mmol/L)	8.90 (6.38, 13.32)	8.80 (6.70, 13.36)	9.64 (6.98, 14.46)	0.010
Glucose 120 min (mmol/L)	16.80 (12.80, 20.70)	16.80 (12.80, 20.60)	16.80 (13.20, 20.90)	0.286
Insulin at 0 min (µIU/mL)	4.59 (2.21, 8.62)	6.05 (3.15, 11.40)	6.75 (3.56, 11.69)	0.158
Insulin at 120 min (µIU/mL)	23.92 (11.98, 43.14)	30.84 (17.11, 59.50)	31.90 (17.60, 57.94)	0.002
HOMA-IR	1.86 (0.87, 3.69)	2.64 (1.36, 4.91)	3.05 (1.60, 5.38)	0.158
HbA1c (%)	9.80 (7.60, 12.20)	10 (7.90, 11.90)	10.10 (8.20, 12.10)	0.174
TC (mmol/L)	4.48 (3.76, 5.30)	4.72 (3.93,5.56)	4.97 (4.21,5.94)	<0.001
LDL-C (mmol/L)	3.08 (2.39,3.75)	3.27 (2.56,4.02)	2.98 (2.33,3.79)	0.269
baPWV (m/s)	1306.50 (1190.13,1479.00)	1541.50 (1311.50,1798.00)	1540.25 (1349.00,1799.63)	<0.001

Data is described as median (interquartile range) or n (%). Linear regression analysis was used for the continuous variables (age, duration, diastolic blood pressure, systolic blood pressure, BMI, hip circumference, glucose at 0 min, glucose at 120 min, insulin at 0 min, insulin at 120 min, HOMA-IR, glycosylated hemoglobin, TC, LDL-C, and baPWV). Chi-square test was performed for the categorical variable(gender). baPWV: brachial-ankle pulse wave velocity; TC: total cholesterol; HOMA-IR: homeostatic model assessment for Insulin resistance

## Comparison of the prevalence of arteriosclerosis

**Fig. 1:**
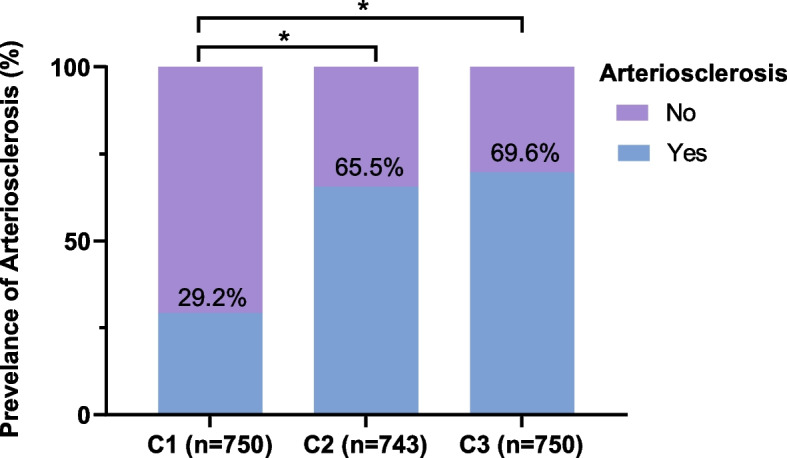
Comparison of the prevalence of arteriosclerosis among the CMI groups.  Patients in Groups C2 and C3 exhibited a significantly greater occurrence of arteriosclerosis than did those in Group C1 ( *P*  < 0.05; Fig.  1). * indicates *P*  < 0.05

As shown in Table 2, 29.2% of the individuals in Group C1, 65.5% of the patients in Group C2, and 69.6% of the patients in Group C3 had arteriosclerosis. A significant trend was evident, showing an increase in the prevalence of arteriosclerosis corresponding to the CMI. Notably, Groups C2 and C3 exhibited a significantly greater occurrence of arteriosclerosis than did Group C1 (*P* < 0.05; Fig. 1). Additionally, Cramer’s V coefficient was utilized to assess the relationship between the CMI and arteriosclerosis, revealing a moderately strong positive correlation (Cramer’s V = 0.365, *P* < 0.001).

**Table 2:** Comparative analysis of the prevalence of arteriosclerosis

Arteriosclerosis	C1 (*n* = 750)	C2 (*n* = 743)	C3 (*n* = 750)	χ^2^	* P *
Yes	219 (29.2%)	487 (65.5%)	522 (69.6%)	299.33	< 0.001
No	531 (70.8%)	256 (34.5%)	228 (30.4%)		

### Correlations between the CMI and clinical parameters

Spearman’s correlation analysis revealed that the CMI was positively correlated with blood pressure (*r* = 0.74, for systolic blood pressure [*P* < 0.001] and *r* = 0.19 for diastolic blood pressure [*P* < 0.001]; Table 3). Additionally, the CMI was positively correlated with BMI (*r* = 0.31, *P* < 0.001) and hip circumference (*r* = 0.22, *P* < 0.001). Notably, baPWV was found to be positively related to the CMI (*r* = 0.32, *P* < 0.001). Although there were significant correlations between the CMI and blood biochemical indicators (glucose at 0 min, insulin at 0 min, insulin at 120 min, HOMA-IR, and TC levels, all *P* < 0.05), the strength of the correlations was comparatively low (*r* = 0.08–0.21).

**Table 3:** Correlation analysis between the CMI and various clinical parameters

Variables	CMI	
	r	* P *
Age	0.03	0.123
Duration	-0.02	0.385
Diastolic blood pressure	0.19	< 0.001
Systolic blood pressure	0.74	< 0.001
BMI	0.31	< 0.001
Hip circumference	0.22	< 0.001
Glucose at 0 min	0.08	< 0.001
Glucose at 120 min	0.01	0.595
Insulin at 0 min	0.18	< 0.001
Insulin at 120 min	0.16	< 0.001
HOMA-IR	0.21	< 0.001
HbA1c	0.04	0.095
TC	0.18	< 0.001
LDL-C	-0.03	0.178
baPWV	0.32	< 0.001

### Association between the CMI and arteriosclerosis

Logistic regression analysis was performed to evaluate the association between the CMI and the occurrence of arteriosclerosis by constructing three models. In Model 1, we adjusted for sex and determined that the OR of the CMI for the development of arteriosclerosis was 1.97(95%CI: 1.76–2.20, *P* < 0.001; Table 4). Compared to the C1 group, the C2 group and C3 group had a greater risk for developing arteriosclerosis (OR = 4.64[95% CI: 3.73–5.77, *P* < 0.001] and OR = 5.63[95%CI: 4.51–7.03, *P*<0.001], respectively). Next, diastolic blood pressure, systolic blood pressure, BMI, and hip circumference were included in Model 2. After considering these covariates, the OR was 1.85 for continuous CMI values (95%CI: 1.65–2.08, *P* < 0.001). Furthermore, Model 3 was adjusted for additional covariates (glucose at 1 min, insulin at 120 min, and TC) exhibited similar trends for continuous (OR = 1.87, 95%CI: 1.66–2.10, *P* < 0.001) and categorical CMI values (OR = 4.55 for Group C2, *P* < 0.001; OR = 5.56 in Group C3, *P* < 0.001).

**Table 4:** Multivariable logistic regression for the association between the CMI and arteriosclerosis

Variables	Model 1		Model 2		Model 3	
	OR (95%CI)	*P*	OR (95%CI)	*P*	OR (95%CI)	*P*
**CMI (continuous)**	1.97 (1.76, 2.20)	<0.001	1.85 (1.65,2.08)	<0.001	1.87 (1.66, 2.10)	<0.001
**CMI (categorical)**						
C1	Reference		Reference		Reference	
C2	4.64 (3.73, 5.77)	<0.001	4.59 (3.61, 5.83)	<0.001	4.55 (3.57, 5.81)	<0.001
C3	5.63(4.51, 7.03)	<0.001	5.45 (4.26, 6.98)	<0.001	5.56 (4.32, 7.17)	<0.001

Odds ratios (*OR*) and *95%CI* were evaluated using a logistic regression model. C1: the lowest tertile of CMI, C2: the 2nd tertile of CMI, C3: the highest tertile of CMI

Model 1: Adjusted for gender

Model 2: Model 1+ adjusted for diastolic blood pressure, systolic blood pressure, BMI, hip circumference.

Model 3: Model 2+ adjusted for glucose at 0 min, insulin at 120 min, and TC

### Stratified analysis of the CMI and arteriosclerosis

**Fig. 2:**
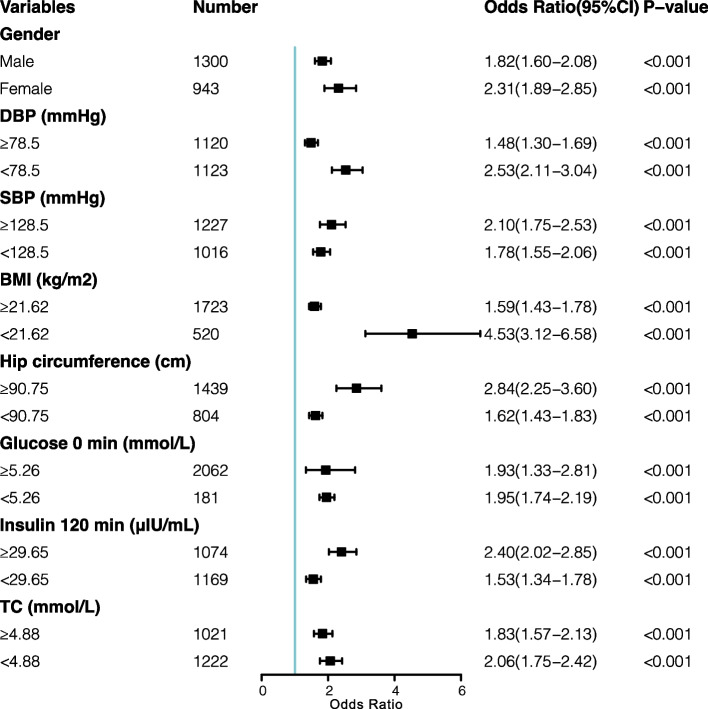
Subgroup analyses of the association between the CMI and Arteriosclerosis. The subgroup analyses were based on the following predetermined cutoff values: diastolic blood pressure (78.50 mmHg), systolic blood pressure (128.50 mmHg), BMI (21.62 kg/m²), hip circumference (90.75 cm), fasting glucose level (5.26 mmol/L), insulin level at 120 minutes (29.65 μIU/mL), and total cholesterol (4.88 mmol/L). CI: confidence interval; OR: odds ratio

As shown in Fig. 2, the relationship between the CMI and arteriosclerosis was assessed by stratifying covariates, including diastolic and systolic blood pressure, BMI, hip circumference, fasting glucose level, insulin level at 120 min, and TC level. The results demonstrated that the CMI was an independent risk factor for an increased incidence of arteriosclerosis across all subgroups (all *P* < 0.001). Interestingly, the association was notably stronger in patients with a BMI below 21.62 kg/m (OR = 1.59, 95%CI: 1.43–1.78) than in those with a BMI of 21.62 kg/m² or higher (OR = 4.53, 95%CI: 3.12–6.58), possibly influenced by the smaller sample size in the lower BMI group.

## Discussion

The findings of this study underscore the intricate link between the CMI and arteriosclerosis in patients with T2DM. Our data revealed a significant positive correlation between the CMI and the development of arteriosclerosis, which was robust even after adjusting for traditional risk factors. This association aligns with previous studies that have revealed the CMI to be a predictor of cardiovascular events in various populations [10, 11]. However, our research extends this knowledge by specifically elucidating the relationship within the T2DM cohort, a group at an inherently higher risk of cardiovascular complications.

Based on the CMI, the patients were categorized into three groups. The C3 group (CMI > 1.355) exhibited greater diastolic blood pressure, systolic blood pressure, BMI, hip circumference, glucose (0 min), insulin (120 min), TC and baPWV values. These findings suggest that the CMI serves as an effective marker for distinguishing T2DM patients with arteriosclerosis from those without arteriosclerosis.

An increased CMI poses a risk for individuals with T2DM and is recognized as a detrimental factor. To effectively prevent the onset of T2DM, appropriate measures should be implemented to mitigate the escalation of the CMI from low to high values [15, 16]. Additionally, the CMI exhibited positive correlations with diastolic blood pressure, systolic blood pressure, BMI, hip circumference, glucose (at 0 min), insulin (at 0 min), insulin (at 120 min), HOMA-IR, TC and PWV values. These findings suggest that the CMI may exert diverse influences on various cardiovascular disease risk factors. Specifically, in obese patients, the CMI might be more directly associated with weight and insulin resistance [17, 18]. In hypertensive patients, the CMI may be related to vascular function [19]. In patients with diabetes, the CMI may be associated with insulin resistance [20]. The group with the highest CMI, referred to as Group C3, exhibited greater diastolic blood pressure, systolic blood pressure, BMI, hip circumference, fasting glucose, 120-minute postload insulin, TC, and baPWV values. These findings suggest that the CMI is an effective marker for distinguishing T2DM patients with arteriosclerosis from those without arteriosclerosis.

Further analysis of the different subgroups revealed a significant correlation between the CMI and an increased incidence of arteriosclerosis in each subgroup. As the CMI increased, so did the prevalence of arteriosclerosis, with an apparent trend across the patient groups. These findings indicate that the CMI could be a key marker for predicting the risk of arteriosclerosis. Clinically, this means that by monitoring changes in the CMI, doctors can detect and act to prevent arteriosclerosis. It is also necessary to create specific prevention and treatment strategies based on the CMIs of different patient subgroups to mitigate the risk of arteriosclerosis. We constructed three models for multivariate regression analysis. In the analysis stratified by the CMI, after adjusting for other variables, the risk of arteriosclerosis in the C2 group was 4.6 times greater than that in the C3 group, and the risk was 5.1 times greater than that in the C1 group. These results suggest that CMI stratification is a valuable predictor of arteriosclerosis risk. Moreover, our study examined the impact of other covariates on arteriosclerosis risk. In Model 3, factors such as age, sex, hypertension, and hyperlipidemia were strongly associated with arteriosclerosis risk.

The biological plausibility of the CMI as a marker for arteriosclerosis can be understood through the lens of insulin resistance [18] and hyperglycemia [21], which are hallmarks of T2DM. These metabolic abnormalities contribute to the endothelial dysfunction [22], oxidative stress [23], and inflammatory processes[24] that are fundamental to the development of arteriosclerosis. Furthermore, the components of the CMI, which include measures of obesity and dyslipidemia, are known contributors to vascular pathology. Hence, our findings suggest that the CMI captures the cumulative effect of these metabolic derangements on arterial health.

However, our research is not without limitations. The design of the study precludes causal inferences. While we demonstrated a correlation, longitudinal studies are necessary to confirm whether changes in the CMI precede the progression of arteriosclerosis. Additionally, our study population may not be representative of all patients with T2DM, and our findings may not be generalizable to other cohorts, particularly those with varying durations of diabetes or levels of glycemic control. To delve deeper into the relationship between the CMI and arteriosclerosis, additional research and experimentation are necessary. We could investigate how the CMI is related to the mechanism of arteriosclerosis or examine the outcomes in patients with varying CMIs undergoing identical treatments.

In conclusion, our findings strongly support the use of CMI stratification as an important predictor of arteriosclerosis risk. These findings also highlight the need to consider other covariates for accurate risk assessment, which has significant implications for the prevention and treatment of arteriosclerosis. Further longitudinal studies are required to establish the prognostic value of the CMI in predicting cardiovascular events in patients with T2DM.

## Data Availability

Data sets generated during the current study are available from the corresponding author on reasonable request.

## Abbreviations

DM: Diabetes mellitus
T2DM: Type 2 diabetes mellitus
CMI: Cardiometabolic index
MMC: National Metabolic Management of Metabolic Center
WHtR: Waist-to-height ratio
HDL-C: High-density lipoprotein
baPWV: Brachial-ankle pulse wave velocity
HOMA-IR: Homeostatic model assessment for Insulin resistance
ORs: Odds ratios
CI: Confidence interval
